# Management of Meningitis Caused by Multi Drug-Resistant *Acinetobacter Baumannii*: Clinical, Microbiological and Pharmacokinetic Results in a Patient Treated with Colistin Methanesulfonate

**DOI:** 10.4084/MJHID.2015.055

**Published:** 2015-10-11

**Authors:** Elisabetta Schiaroli, Maria Bruna Pasticci, Maria Iris Cassetta, Stefania Fallani, Corrado Castrioto, Matteo Pirro, Andrea Novelli, Lucia Henrici De Angelis, Marco Maria D’Andrea, Maria Lina Mezzatesta, Franco Baldelli, Antonella Mencacci

**Affiliations:** 1 Unit of Infectious Diseases, Department of Medicine, University of Perugia, Perugia, Italy; 2 Department of Health Science, University of Florence, Florence, Italy; 3 Unit of Neurosurgery, Hospital Santa Maria della Misericordia, Perugia, Italy; 4 Unit of of Internal Medicine, Department of Medicine, University of Perugia, Perugia, Italy; 5 Department of Medical Biotechnologies, University of Siena, Siena, Italy; 6 Department of Biomedical and Biotechnological Sciences, University of Catania, Catania Italy.; 7 Unit of Microbiology, Department of Experimental Medicine and Biochemical Sciences, University of Perugia, Perugia, Italy

## Abstract

This paper reports on a 71- year-old Caucasian male who underwent neurosurgery for an oligodendroglioma, followed by a cranial-sinus fistula and cerebrospinal fluid rhinorrhea. The clinical course was complicated due to an extensively drug-resistant *Acinetobacter baumannii* meningitis. The patient was treated with colistin methanesulfonate, intrathecal for 24 days and intravenous for 46 days. In addition, the patient received meropenem and teicoplanin to treat a urinary tract infection and a bacterial aspiration pneumonia. Cerebrospinal fluid trough colistin levels resulted above the MIC of *A. baumannii*. Colistin cerebrospinal fluid concentration did not increase over the treatment period. Meningitis was cured and *A. baumannii* eradicated. No side effects from the antimicrobial therapy were observed. In conclusion, this case highlights the issues in treating infections caused by resistant Gram negative bacteria and supports previous findings on the efficacy, pharmacokinetic and tolerability of intravenous and intrathecal colistin treatments.

## Introduction

Over the last decade, extensively drug-resistant Gram-negative bacteria, including *Acinetobacter baumannii*, have become a serious cause of hospital-acquired infections. *A. baumannii* has also emerged as a cause of central nervous system (CNS) infections, which are often associated with the use of external cerebrospinal fluid (CSF) catheters.^1^–^3^ The treatment of these infections can often be extremely complex due to antimicrobial resistance and the inadequate antimicrobial concentration at the site of infection.^1^–^6^ The increased rate of infections due to multi drug-resistant Gram-negative bacteria has been reported to lead to a revival in the use of “forgotten” antibiotics, such as colistin.^4^,^5^

Colistin, a polymyxin antibiotic that is administered intravenously (IV) as colistin methanesulfonate, is a prodrug that is converted in vivo and in vitro into its active form colistin.^4^,^6^ Colistin methanesulfonate and colistin poorly cross the brain blood barrier,^4^,^6^ thus in order to treat CNS infections, colistin methanesulfonate needs to be administered either intrathecally (IT) or intraventricularly (IVT).^3^,^6^ The authors report on a case of meningitis caused by multi-drug resistant *A. baumannii* treated with IV and IT colistin.

## Case Report

A 71-year-old Caucasian male was admitted to our hospital with cerebrospinal fluid rhinorrhea one month after having undergone neurosurgery for an oligodendroglioma. Ten days after admission (Table 1), the patient manifested acute meningitis caused by methicillin-resistant *Staphylococcus aureus* (MRSA) and *Corynebacterium striatum*, treated with vancomycin IV 750 mg TID and imipenem IV 500 mg QD. At the same time, the cranial-sinus fistula was repaired. The clinical course was complicated by pneumonia and acute respiratory insufficiency requiring assisted mechanical ventilation (Table 1). A week later, the patient was extubated and re-admitted to the floor. The following day, the patient manifested a low-grade fever and blood tests evidenced increased leukocyte and neutrophil counts; whereas the C-reactive protein (C-RP) and erythrocyte sedimentation rate (ESR) values were 15.4 mg/dL (normal <0.5mg/dL) and 8 mm 1st h (normal 1–30), respectively. Due to a persistent drowsiness and a suspected hydrocephalus, an external CSF lumbar catheter was positioned. The CSF from the catheter resulted having normal cell and glucose values, and the microbiological investigations were negative. Additionally, *Enterobacter cloacae* urinary infection was treated with meropenem (Table 1). Five days later, the patient’s temperature rose to 38.8°C, the leukocyte, neutrophil, C-RP and ERS values also increased, the patient manifested a more depressed level of consciousness and the patient complained of neck stiffness. Simultaneous CSF findings from the lumbar catheter were consistent with acute Gram-negative bacterial meningitis.^7^ In addition, the SeptiFast real-time PCR (SF) (Roche Diagnostics, Monza, Italy)^8^ performed on the CSF sample from the lumbar catheter resulted positive for *A. baumannii* and *K. pneumoniae*, while the CSF mass spectrometry by matrix-assisted laser desorption/ionization time-of-light (MALDI-TOF) (Bruker Daltonics, Bremen, Germany)^9^ was negative. CSF culture yielded *A. baumannii* and a few colonies of *K. pneumoniae*, both susceptible only to colistin. Antimicrobial therapy was administrated : intravenous colistin methanesulfonate 4.500.000 International Unit (IU) (equal to 150 mg of colistin based activity) BID (infused over 30′), meropenem 2 g TID, rifampin 600 mg OD and teicoplanin 600 mg OD after the loading dose plus colistin methanesulfonate IT 125.000 IU (equal to 4.16 mg of colistin based activity) a day.^3^,^10^ Rifampin had to be discontinued soon after due to an allergic reaction. Two days later, after three doses of IV colistin and a single dose of IT colistin, a repeated culture of CSF, from both the lumbar catheter and rachicentesis, evidenced *A. baumannii*. Whenever IT colistin was administered (range of time ± 4h), the catheter was kept closed for 3 hours after. CSF samples for laboratory investigations and concentrations were collected from the lumbar catheter before colistin was administered. Colistin concentrations were evaluated on samples (stored at −20°C until testing) using an HPLC method having fluorimetric detection and netilmicin as an internal standard. Linear calibration curves were obtained by the concentrations of colistin sulfate from 0.30 to 5.0 mg/L in plasma.^11^ On day four of therapy, the patient was without fever, CSF cell count was decreased, and the culture resulted negative. After a total of 24 days of therapy, the lumbar catheter was removed, while a lumbar-peritoneal catheter was positioned to treat a hydrocephalus that had developed. Results of CSF findings are reported in Table 2. IT colistin was discontinued while IV colistin, meropenem, and teicoplanin were continued for a further 22 days, followed by meropenem 3 g and oral doxycycline 200 mg per day for another 11 days. During this period, the patient was without fever but multiple episodes of acute respiratory insufficiency occurred, along with alternatively reduced or increased neutrophils values, C-RP values, and lung infiltrates. Repeated bronchoscopic aspirations were performed, and a percutaneous endoscopic gastrostomy (PEG) was positioned (Table 1). Repeated respiratory secretion cultures evidenced MRSA and *K. pneumoniae* resistant to colistin, but fosfomycin susceptible (Table 1). Despite fosfomycin therapy, the patient had a fatal episode of acute respiratory insufficiency leading to his death.

## Discussion

Over the last decade, the frequency of CNS infections caused by Gram-negative bacteria has increased from 12–27% of cases,^1^–^3^ as well as meningitis caused by *A. baumannii*.

In our patient, clinical and microbiological findings supported a diagnosis of hospital acquired *A. baumannii* meningitis.^7^ In fact, 1) *A. baumannii* was detected by culture and SF in CSF samples obtained from both rachicentesis and the lumbar catheter on the third day of treatment; 2) *A. baumannii* DNA was detected by SF (data not shown) in the CSF from day 12 of treatment; 3) airways were colonized/infected with *K. pneumoniae*, leading us to deduce that the CSF could have been contaminated with this microorganism during collection.^3^,^7^ To this regard, it is important to report that *K. pneumoniae* was cultured with *A. baumannii* from the CSF taken on both day one when the patient had acute bacterial meningitis and on day 17 of treatment when there was clinical improvement and CFS laboratory parameters resulted normalized. *Pseudomonas aeruginosa* detected from the lumbar catheter was considered of no clinical relevance, given an absence of symptoms and CFS abnormalities.^7^

Culture results, SF, and MALDI-TOF tests were performed. Microbial culture is still considered the reference method for infection diagnosis. SF test is a molecular based method used to detect bacteria from blood, but it has also been applied to samples different from blood.^8^ One of the advantages of SF over culture is that the results of this test can be available in less than 6 h, allowing for a prompt and more accurate empiric therapy. Moreover, SF has a high sensitivity for identifying microbial DNA in patients receiving antimicrobial therapy.^8^ However, the clinical significance of blood microbial DNA, even when patients are septic in the absence of microorganism growth, is not well defined.^12^ As well, there are limited data on the reliability of performing SF on other biological samples.^8^ MALDI-TOF is considered a reliable, rapid method for identifying bacterial strains from colonies on solid culture media^9^ and has also been employed to analyze clinical specimens such as urine and CSF for direct bacterial identification. Nevertheless, for our patient, the best results were obtained when the bacterial concentration in the sample was ≥105CFU/mL.^9^

Colistin is defined as a concentration dependent bactericidal antibiotic, therefore, according to Hara GH et al., peak levels seem to be more predictive of clinical efficacy.^4^ In vitro and in vivo animal studies suggest that the area under the curve AUC/MIC and Cmax/MIC ratio is the best predictor of antibacterial activity. However, the pharmacodynamic parameters that best predict efficacy are not well defined.^5^,^6^ Overall, it has been suggested to maintain steady state levels ≥2 mg/L for effective therapy.^5^,^6^

Considering the in vitro antimicrobial susceptibility of *A. baumannii* isolate and the poor capacity of colistin methanesulfonate to cross the blood brain barrier, colistin methanesulfonate was administered both intravenously and intrathecally without a loading dose.^3^,^10^ Overall, the treatment resulted being both effective and well tolerated.

In our patient, only trough CSF values were obtained, and on 3 different days values below 2 mg/L were observed. The ratio between CSF concentration and *A. baumannii* MIC ranged between 2 and 70. This broad range could have been due to the different collection times of CSF and/or CSF efflux fluctuations through the external drainage.^6^ Overall, the CSF colistin concentration did not increase over time, mirroring results by Imberti et. al.^6^ Regarding the Colistin blood levels without a loading dose, values above ≥2 mg/L were registered on day 5 of therapy and a lower colistin concentration in the respiratory secretions most likely favored the selection of Colistin hetero-resistant *K. pneumoniae* isolates.

Considering in vitro susceptibility results of K. pneumoniae isolates to fosfomycin, it is plausible that the variable results reported from our laboratory were due to MIC being close to the susceptibility break point. When these *K. pneumoniae* isolates were evaluated at a reference laboratory they were reported as susceptible to fosfomycin, suggesting that laboratory fluctuation could have induced variable susceptibility results. Furthermore, PFGE analysis of these isolates showed an analogous pattern which was similar to the the international blaKPC-3-positive ST258b hybrid clone (data not shown).

It has been suggested that CSF catheters need to be removed in order to achieve recovery from a CNS infection. However, the exact time of CSF catheter removal has yet to be clearly defined.^3^,^10^ In our case, the infection was controlled, and CSF cultures were negative after 4 days of treatment, thus, we decided to keep the external lumbar catheter in place. It was removed only after the meningitis was cured, and a lumbar-peritoneal derivation could be placed without a high risk of relapse.

In conclusion, this case highlights the issues involved in treating infections caused by drug-resistant Gram-negative bacteria and supports previous findings on the efficacy, pharmacokinetics and tolerability of intravenous and intrathecal Colistin treatments.

## Figures and Tables

**Table 1:** Clinical course and microbiological findings.

Time	Diagnosis	Body temperature	Blood WBCs ×10^3^ (N%)	CSF microbiology	Respiratory secretions microbiology	Invasive procedure/s	Antimicrobial therapy
Day 10	Bacterial meningitis	38.8°C	8.43 (82%)	*MRSA+ *C. striatum*	*MRSA	Cranial-sinus fistula repair CSF lumbar catheter	Vancomycin 750 mg q8h Imipenem 500 mg q6h
Day 32	Pneumonia+ Respiratory insufficiency	38.0°C	10.7 (87%)		*MRSA	Assisted ventilation	Linezolid 600 mg q12h
Day 43	°UTI+ Pneumonia	37.6°C	9.74 (89%)	**Negative	MRSA+ *Citrobacter koseri*+ §*K. pneumoniae*	CSF lumbar catheter Bronchoscopic aspiration	Linezolid 600 mg q12h Meropenem 2 g q8h
Day 58	Pneumonia+ Bacterial meningitis	38.8°C	9.77 (95%)	**A*. baumannii+* **K. pneumoniae		Rachicentesis	Meropen 2 g q8h Teicoplanin 600 mg q24h Colistin 4.500.000 IU q12h Colistin (IT) 125.000 IU q24
Day 63	Pneumonia+ Bacterial meningitis	<37.0°C	5.90 (69%)	Negative			Meropen 1 g q8h Teicoplanin 600 mg q24h Colistin 4.500.000 IU q12h Colistin (IT) 125.000 IU q24
Day 74	Pneumonia+ Recurrent respiratory insufficiency	<37.0°C	4.64 (70%)	**Negative	MRSA+ ¶*K. pneumoniae*	Bronchoscopic aspiration	Meropen 1 g q8h Teicoplanin 400 mg q24h Colistin 3.000.000 IU q12h
Day 81	Clinical improvement Meningitis cured	<37.0°C	4.96 (73%)		Negative	CSF lumbar catheter removed CSF lumbar-peritoneal catheter positioned	Meropen 1 g q8h Teicoplanin 400 mg q24h Colistin 3.000.000 IU q12h
Day 94	Pneumonia+ Recurrent respiratory insufficiency	<37.0°C	7.87 (78%)		MRSA+ #*K. pneumoniae* colistin resistant	Repeated bronchoscopic aspiration	Meropenem 1 g q8h Doxicicline 100 mg q12h (OS)
Day 105	Pneumonia+ Recurrent respiratory insufficiency	<37.0°C	7.49 (82%)		#*K. pneumoniae* colistin resistant	Repeated bronchoscopic aspiration Percutaneous gastrostomy	Meropenem 1g q8h Doxicicline 100 mg q12h
Day 108	Pneumonia+ Recurrent respiratory insufficiency	<37.0°C	5.89 (75%)		#*K. pneumoniae* colisitn resistant *+ A. baumannii* multi susceptible	Repeated bronchoscopic aspiration	fosfomycin 2g q6h

# CSF collected from the nose;

* CSF collected from spinal tap;

** CSF collected from the lumbar drain;

° UTI=Urinary tract infection (pyuria and urine culture positive for *Enterobacter cloacae*;

**,§ colistin MIC 0.125 mg/L;

¶ colistin MIC >4mg/L;

IT= Intratecal, WBCs=white blood cells; MRSA=methicillin resistant *Staphylococcus aureus*. In vitro susceptibility tests were performed with the BD Phoenix automated (BD Diagnostic Systems, Sparks, MD), E-test method (bioMerieux, Durham, NC). Break points for susceptibility were defined according to the EUCAST document.

**Table 2:** Cerebrospinal fluid findings

Date	Sample	Cells/mm^3^	Ratio CSF glucose/Blood glucose	Proteins mg/dL	Agar culture	Broth culture	Colistin levels mg/L
23/11	CSF from catheter&	8100	1/132	361	*^A. baumannii*, *K. pneumoniae (few colonies)	*^A. baumannii*, *K. pneumoniae	<0.15
25/11	CSF from catheter&	400	8/146	177	*A. baumannii*	*A. baumannii*	2.40
25/11	CSF from spinal tap&	1900	8/146	152	*A. baumannii*	*A. baumannii*	0.28
26/11	CSF from catheter	8560	10/139	187	Negative	Negative	7.10§
27/11	CSF from catheter	3000	20/124	253	Negative	Negative	0.25§§
28/11	CSF from catheter	172	31/110	180	Negative	Negative	6.55
5/12	CSF from catheter	64	33/not done	248	Negative	Negative	8.40
10/12	CSF from IT catheter&	50	45/121	380	Negative	**K. pneumoniae	1.28
17/12	CSF from spinal tap	Absent	50/118	111	Negative	Negative	
17/12	Catheter	Not applicable	Not applicable	Not applicable	Negative	*P. aeruginosa*	

^ *A. baumannii* colistin MIC 0.125 mg/L,

* *K. pneumoniae* susceptible to colistin (MIC=0.125 mg/L), resistant to fosfomycin (MIC ≥256 mg/L),

** *K. pneumoniae* susceptible to colistin (MIC=1 mg/L) and fosfomycin (MIC ≤16 mg/L),

§ 0.38mg/L,

§§ 2.66mg/L values of simultaneous plasma levels,

& same results with the real-time PCR SeptiFast test. CSF samples for microbiologic investigations and colistin concentrations were collected from the lumbar catheter before colistin administration, occurring each day with a range of ± 4 h. Samples collected for colistin concentrations were immediately stored at −20°C until testing.
